# Prevalence, diversity, and host associations of *Bartonella* strains in bats from Georgia (Caucasus)

**DOI:** 10.1371/journal.pntd.0005428

**Published:** 2017-04-11

**Authors:** Lela Urushadze, Ying Bai, Lynn Osikowicz, Clifton McKee, Ketevan Sidamonidze, Davit Putkaradze, Paata Imnadze, Andrei Kandaurov, Ivan Kuzmin, Michael Kosoy

**Affiliations:** 1 National Center for Disease Control and Public Health, Tbilisi, Georgia; 2 Ilia State University, Tbilisi, Georgia; 3 Centers for Disease Control and Prevention, Division of Vector-Borne Disease, Fort Collins, Colorado, United States of America; 4 Department of Biology, Colorado State University, Fort Collins, Colorado, United States of America; 5 Institute of Zoology, Ilia State University, Tbilisi, Georgia; 6 Department of Pathology, University of Texas Medical Branch, Galveston, Texas, United States of America; Institut Pasteur, FRANCE

## Abstract

*Bartonella* infections were investigated in seven species of bats from four regions of the Republic of Georgia. Of the 236 bats that were captured, 212 (90%) specimens were tested for *Bartonella* infection. Colonies identified as *Bartonella* were isolated from 105 (49.5%) of 212 bats Phylogenetic analysis based on sequence variation of the *glt*A gene differentiated 22 unique *Bartonella* genogroups. Genetic distances between these diverse genogroups were at the level of those observed between different *Bartonella* species described previously. Twenty-one reference strains from 19 representative genogroups were characterized using four additional genetic markers. Host specificity to bat genera or families was reported for several *Bartonella* genogroups. Some *Bartonella* genotypes found in bats clustered with those identified in dogs from Thailand and humans from Poland.

## Author summary

Bacteria of the genus *Bartonella* parasitize erythrocytes and endothelial cells of a wide range of mammals and recently were reported in bats from Africa, Asia, America, and northern Europe. A human disease case in the USA was associated with a novel *Bartonella* species, which later was identified in bats in Finland. This human case has demonstrated the zoonotic potential of bat-borne *Bartonella* and underscores the need for extended surveillance and studies of these pathogens. The present work assesses prevalence and diversity of *Bartonella* in bats in the country of Georgia (southern Caucasus), characterizes reference strains representing diverse genogroups by variation of genetic loci, and evaluates the links between identified *Bartonella* genogroups and bat hosts. Importantly, some *Bartonella* genotypes found in bats were close or identical to those identified in dogs and humans. The data indicate that the public health impact of *Bartonella* carried by bats should be investigated.

## Introduction

Bats (Order: Chiroptera) are hosts of a wide range of zoonotic pathogens. Their significance as reservoirs of emerging infectious diseases, predominantly of viral origin, has been increasinglyecognized during recent decades [1,2]. In contrast, the study of bacterial infections in bats hasprogressed more slowly [3]. Bacteria of the genus *Bartonella* are small and slow-growing Gram-negative aerobic bacilli. These bacteria parasitize erythrocytes and endothelial cells of a wide range of mammals. During the last six years, diverse *Bartonella* strains were identified in bats from Europe [4–6], Africa [7–12], Asia [13,14], and Latin America [15–19]. Recent studies have demonstrated significant patterns of evolutionary codivergence among bats and *Bartonella*, demonstrating that strains of *Bartonella* in bats tend to cluster according to bat families, superfamilies, and suborders [20,21]. Host specificity and codivergence have also been documented in rodent-associated *Bartonella* strains [20,22] and bat-associated *Leptospira* strains [23]. Despite their apparent host associations, *Bartonella* spp. can spillover into phylogenetically distant hosts, including humans [24,25]. A recent human case of endocarditis in the US Midwest was associated with a novel *Bartonella* species (*B*. *mayotimonensis*; [26]), which later was isolated in bats in Europe [5]. This human case has demonstrated the zoonotic potential of bat-borne *Bartonella* and underscores the need for extended surveillance and studies of these pathogens.

The goal of the present work was to identify prevalence and diversity of *Bartonella* in bats in theRepublic of Georgia (southern Caucasus) with the following objectives: 1) to compare prevalence of *Bartonella* infection in diverse bat species from different geographic locations within Georgia; 2) to determine the genotypes of obtained strains by variation in *gltA* sequences, a gene commonly used for discrimination of *Bartonella* species; 3) to characterize reference strains representing diverse genogroups by variation of multiple genetic loci; and 4) to evaluate the links between identified *Bartonella* genogroups and bat hosts.

## Materials and methods

### Ethics statement

All animal work has been conducted according to relevant NCDC, national, and international guidelines.

### Capture and sample collection

Bats were collected from two distinct parts of Georgia in June 2012. Four locations are situated in Eastern Georgia: three sites in the Kakheti region near Davit Gareja, one site in the Kvemo Kartli region in Gardabani district. The other four locations are in Western Georgia: two sites in the Samegrelo-Zemo Svaneti region (Martvili district and Chkhrotsku district) and two sites in the Imereti region (Terjola district and near Tskaltubo town). The number of captured bats from each site is shown in Table 1.

**Table 1:** Prevalence of *Bartonella* infection across bat species, collection locations, and sexes. Confidence intervals were calculated using the Agresti-Coull method.

**Species**	**Family**	**Captured**	**Tested**	**Positive**	**Positive (%)**	**95% CI**	**Coinfections**
*Eptesicus serotinus*	Vespertilionidae	20	20	4	20.0	[7.5, 42.2]	0
*Miniopterus schreibersii*	Miniopteridae	29	27	24	88.9	[71.1, 97]	7
*Myotis blythii*	Vespertilionidae	75	67	32	47.8	[36.2, 59.5]	3
*Myotis emarginatus*	Vespertilionidae	42	38	15	39.5	[25.6, 55.3]	1
*Pipistrellus pygmaeus*	Vespertilionidae	13	12	2	16.7	[3.5, 46]	0
*Rhinolophus euryale*	Rhinolophidae	29	26	18	69.2	[49.9, 83.7]	2
*Rhinolophus ferrumequinum*	Rhinolophidae	27	22	10	45.5	[26.9, 65.4]	3
**Location**	**Habitat** Log Lat	**Captured**	**Tested**	**Positive**	**Positive (%)**	**95% CI**	**Species distribution**
Davit Gareja, Tetri Senakebi	41.53603 45.257048	25	21	11	52.4	[32.4, 71.7]	13 ME, 12 RF
Davit Gareja, John the Baptist Cave	41.298611 45.704722	25	24	15	62.5	[42.6, 78.9]	25 MB
Davit Gareja, Lavra	41.447472 45.376472	8	6	1	16.7	[1.1, 58.2]	1 MB, 7 RF
Davit Gareja, total		58	51	27	52.9	[39.5, 65.9]	26 MB, 13 ME, 19 RF
Gardabani Managed Reserve	41.37699 45.0791	50	46	14	30.4	[19, 44.9]	20 ES, 15 ME, 1 MM, 13 PP, 1 RF
Martvili, Leskhulukhis Cave	42.52927 42.10283	22	21	13	61.9	[40.8, 79.3]	15 RE, 7 RF
Terjola, Dzeveri, Bzebi Restaurant Cave	42.183333 42.983333	20	18	10	55.6	[33.7, 75.5]	5 MS, 15 MB
Tskaltubo, Gliana Cave	42.37302 42.59748	53	48	31	64.6	[50.4, 76.6]	18 MS, 26 MB, 9 RE
Chkhorotsku, Letsurtsume Cave	42.10375 42.32454	33	28	10	35.7	[20.6, 54.2]	6 MS, 8 MB, 14 ME, 5 RE
Western Georgia, total		106	94	51	54.3	[44.2, 64]	29 MS, 49 MB, 14 ME, 14 RE
**Sex**		**Captured**	**Tested**	**Positive**	**Positive (%)**	**95% CI**	
Female		177	160	73	45.6	[38.1, 53.4]	
Male		59	50	30	60.0	[46.2, 72.4]	
**All total**		**236**	**212**	**105**	**49.5**	**[42.9, 56.2]**	

Two positive *Myotis blythii* were not sexed. Species abbreviations: ES–*Eptesicus serotinus*, MB–*Myotis blythii*, ME–*Myotis emarginatus*, MS–*Miniopterus schreibersii* sensu lato, PP–*Pipistrellus pygmaeus*, RE–*Rhinolophus euryale*, RF–*Rhinolophus ferrumequinum*.

Bats were captured manually or using nets from different roosts in caves and buildings (attics, cellars, and monasteries). The list of bat species and the number of animals per roost or colony availablefor sampling was approved by the Ministry of Environmental and Natural Resources Protection ofGeorgia. Species of captured bats were identified based on external morphological characteristics. Captured bats (n = 236) were delivered to the processing site in individual cotton bags where they were processed. Bats were anesthetized with the use of ketamine (0.05–0.1 mg/g body mass) and exsanguinated by cardiac puncture. All bats were sexed and measured. The procedures of handling animals were performed in compliance with the protocol approved by the CDC Institutional Animal Care and Use Committee (protocol 2096FRAMULX-A3). Blood specimens were transported on dry ice to the NCDC Laboratory, Tbilisi where they were stored at -80°C until they were shipped on dry ice to the CDC’s laboratory, Fort Collins, Colorado. Upon arrival at CDC, the samples were stored at -80°C until they were analyzed.

### Culturing

Bat blood was diluted 1:4 in Brain Heart Infusion (BHI) with 5% Fungizone (amphotericin B), and 100μl of the sample was placed on a chocolate agar plate following the protocol published previously [27]. Inoculated plates were incubated at 35°C in a 5% CO2 environment for up to five weeks. Plates werechecked periodically, and bacterial colonies that morphologically resembled those typical for *Bartonella*were passaged onto a new plate to obtain pure cultures. In an attempt to capture possible *Bartonella* coinfections, all morphologically unique colonies growing from a single sample were sub-passaged and sequenced. All resulting isolates were collected in a 10% glycerol solution. Crude DNA extracts were obtained from isolates by heating a heavy suspension of themicroorganisms for 10 minutes at 95°C. Polymerase chain reactions (PCR) with the *gltA* primersBhCS781.p (5’-GGGGACCAGCTCATGGTGG-3’) and BhCS1137.n (5’-AATGCAAAAAGAACAGTAAACA-3’) [28] were performed using PCR Thermal Cycler Dice(Takara Bio Inc., Japan) and C1000 Touch Thermal Cycler (Bio-Rad, Berkeley, CA). Positive (*B*. *doshiae*) and negative (nuclease free water) control samples were included in each PCR assay to evaluate the presence of appropriately sized amplicons and to rule out contamination of reagents, respectively. Positive PCR products were purified using QIAquick PCR purification Kit (Qiagen, Valencia, CA) and sequenced with an ABI 3130 Genetic Analyzer (Applied Biosystems, Foster City, CA). Forward and reverse reads were assembled into consensus sequences with the SeqMan Pro program in Lasergene v. 11 (DNASTAR, Madison, WI).

### Phylogenetic analysis

A BLAST (http://blast.ncbi.nlm.nih.gov/Blast.cgi) search of the GenBank database was performed with all assembled *gltA* sequences to verify their *Bartonella* origin. Positive sequences were aligned with *Bartonella* reference sequences available in GenBank which included sequences obtained from various bats in previous studies. *Brucella abortus* sequence was used as outgroup. Alignment was performed with MAFFT v7.187 using the local, accurate L-INS-i method [29]. The optimal evolutionary model for the aligned sequences was determined by jModelTest2v2.1.6 [30] using Akaike information criterion corrected for finite sample sizes (AICc) for modelselection [31]. For our dataset, the best model was the generalized time-reversible substitution model with four gamma-distributed categories and a proportion of invariant sites (GTR+Γ+I). We implementedthis model for the Bayesian phylogeny of our sequences with BEAST v1.8.3 [32,33]. Since our goal was only to reconstruct the evolutionary topology of the sequences and not any demographic parameters, we assumed a constant population size for all branches. Similarly, we chose a strict molecular clock because the *Bartonella* sequences from Georgian bats were all isolated at the same date and thus could not be used for calibration of another clock model; furthermore, our analysis did not seek to accurately deduce branch times, and the strict clock was adequate. No codon partitioning was used due to the fact that *gltA* sequences represent only a 367 base pair fragment of the entire gene; codon partitioning with limited genetic information can substantially reduce the effective sample size of estimated parameters forseparate codon positions [34]. All priors were kept at the default, diffuse settings (see Appendix) and the number of Markov chain Monte Carlo (MCMC) iterations was set to 1.2E8 with states sampled every 1.2E4 steps. Three independent chains were run and effective sample sizes and convergence ofparameters during MCMC sampling were assessed using Tracer v1.6 [32]. TreeAnnotator was used to find the most probable tree with burning 10% of the initial trees. The selected tree was then visualizedand edited in FigTree v1.4.2 [35]. Sequence alignment with MAFFT and phylogenetic analysis withBEAST were run using XSEDE supercomputing resources [36], accessed through the CIPRES ScienceGateway [37]. A quantitative threshold for demarcation of sequences into genogroups was set at 96%nucleotide identity following recommendations by La Scola et al. proposed for demarcation of *Bartonella* species [38]. Based on this clustering scheme, branches on the phylogenetic tree were collapsed and annotated with the number of sequences included in each genogroup and the range of DNA identity values.

### Multi-locus typing of reference strains

Five genetic loci (*ftsZ*, *gltA*, *nuoG*, *rpoB*, and *groEL*) that have been previously used for bartonellacharacterization [9,39,40] were additionally investigated in 21 isolates representing 19 diverse genogroups identified based on variation of the *gltA* gene. Genogroups Vesp-7, Vesp-13, and Rhin-3 were not analyzed by MLST, while three isolates of Vesp-6 were selected for analysis to examine within-genogroup variation. The primers and cycle conditions used to generate sequences for each loci have been previously published [28,41–44]. Sequences were aligned with those of the reference *Bartonella* species and other *Bartonella* sequences obtained from bats with MAFFT v7.187 using the L-INS-i method [29]. Evolutionary model selection was performed for each marker separately and for the concatenated sequences using jModelTest2 v2.1.6 [30] based on AICc [31]. Again, the best available model for all sequences was GTR+Γ+I. A Bayesian tree was inferred using BEAST v1.8.3 [33] with the same settings and resources as for the *gltA* tree as described above. Separate maximum likelihood gene trees were generated using the GTRCAT model in RAxML [45]. A network phylogeny was created using the NeighborNet algorithm in SplitsTree v4.13.1 [46] and the pairwise homoplasy index [47] was calculated to test for evidence of recombination among genogroups. All unique sequences were uploaded to GenBank with accession numbers KX300105-KX300201 (Table 2).

**Table 2:** GenBank accession numbers for *Bartonella* strains from Georgian bats characterized by MLST.

Isolate B#	Host	*gltA* genotype	*ftsZ* accession	*gltA* accession	*groEL* accession	*nuoG* accession	*rpoB* accession
44718	*Pipistrellus pygmaeus*	Vesp-11	KX300177	KX300179	KX300180	KX300178	KX300181
44617	*Myotis emarginatus*	Vesp-12	KX300148	KX300149	KX300150	KX300151	KX300152
44724	*Myotis emarginatus*	Vesp-3	KX300153	KX300154	KX300155	KX300156	KX300157
44731	*Myotis blythii*	Vesp-1	KX300139	KX300140	KX300141	KX300142	KX300143
44722	*Eptesicus serotinus*	Vesp-2	KX300199	KX300200	KX300201	KX300202	KX300203
44530	*Miniopterus schreibersii*	Mini-1	KX300175	KX300183	KX300184	KX300185	KX300186
44608	*Miniopterus schreibersii*	Mini-1.1	KY679153	KX300195	KX300196	KX300197	KX300198
44599	*Miniopterus schreibersii*	Mini-2	KX300191	KX300192	KY679156	KX300193	KX300194
44593	*Miniopterus schreibersii*	Mini-3	KX300187	KY679154	KX300189	KX300190	KX300188
44602	*Myotis blythii*	Vesp-10	KX300116	KX300117	KX300118	KX300119	KX300120
44715	*Myotis blythii*	Vesp-6	KX300130	KX300131	KY679157	KX300132	KX300133
44719	*Myotis blythii*	Vesp-6	KX300134	KX300136	KX300137	KX300138	KX300135
44711	*Myotis blythii*	Vesp-6	KX300126	KX300127	KY679158	KX300128	KX300129
44591	*Myotis blythii*	Vesp-8	KX300106	KX300107	KX300108	KX300109	KX300110
44544	*Myotis emarginatus*	Vesp-9	KX300176	KX300145	KX300146	KX300144	KX300147
44528	*Rhinolophus euryale*	Rhin-4	KX300105	KX300158	KX300159	KX300160	KX300161
44658	*Rhinolophus ferrumequinum*	Rhin-2	KX300174	KX300165	KX300166	KX300167	KX300170
44552	*Rhinolophus ferrumequinum*	Rhin-5	KX300182	KY679155	KX300162	KX300163	KX300164
44706	*Rhinolophus ferrumequinum*	Rhin-1	KX300168	KX300169	KX300171	KX300172	KX300173
44601	*Myotis blythii*	Vesp-4	KX300111	KX300112	KX300113	KX300114	KX300115
44622	*Myotis blythii*	Vesp-5	KX300121	KX300123	KX300124	KX300125	KX300122

### Accession numbers

Data available from the Dryad Digital Repository: http://dx.doi.org/10.5061/dryad.f0k4j

### Statistical analysis

A logistic model was used to examine important predictors of *Bartonella* prevalence in Georgian bats. For this analysis, we included such variables as bat species, sex, capture location, and capture region. Additional size measurements (weight and forearm length), were collapsed into a single principlecomponent that explained 95% of variation in size. However, bat size was strongly predicted by batspecies (F = 534.6, p-value = 2E-16) and sex (F = 25, p-value = 1.3E-6), so size was not included as acovariate in the global model. Model selection was based on AICc [31]. Additional tests, including Waldtests of fixed effects and calculation of the area under the receiver operating characteristic curve (AUC),were performed on models within two AICc of the top model (ΔAICc) [48,49]. Binomial confidenceintervals for *Bartonella* prevalence among bat species, capture locations, and bat sexes wereapproximated with the Agresti-Coull method [50]. All statistical tests were performed in R [51] andvalues were considered significant for P < 0.05. Additional details of the statistical tests can be found inthe Appendix.

## Results

### Bat species by site

A total of 236 bats were sampled from eight field sites in four regions of Georgia. The sampled batsincluded eight species: common serotine, *Eptesicus serotinus* (Vespertilionidae; n = 20); Schreibers's long-fingered bat, *Miniopterus schreibersii* sensu lato (Miniopteridae; n = 29) [52]; lesser mouse-eared myotis, *Myotis blythii* (Vespertilionidae; n = 75); Geoffroy's myotis, *Myotis emarginatus* (Vespertilionidae; n = 42); whiskered myotis, *Myotis mystacinus* (Vespertilionidae; n = 1); soprano pipistrelle, *Pipistrellus pygmaeus* (Vespertilionidae; n = 13); Mediterranean horseshoe bat, *Rhinolophus euryale* (Rhinolophidae; n = 29); and greater horseshoe bat, *Rhinolophus ferrumequinum* (Rhinolophidae; n = 27). The number of species and specimens obtained varied per site (Table 1).

### Prevalence of *Bartonella* infections in bats

A total of 212 bats of seven species were available for *Bartonella* testing. The amount of blood from thesingle captured *My*. *mystacinus* was not sufficient for culturing. Except for this, bartonellae weresuccessfully cultured from all bat species tested (Table 1). *Bartonella* colonies became visible within 3–28 days after plating. All plates remained free of contamination for the entire five week period and only *Bartonella*-like colonies were observed. Most of the isolated colonies appeared small, circular, and raised, with smooth or rough morphology. The number of *Bartonella*-like colonies observed per plate ranged from 1 colony to “too numerous to count” (TNTC). All the harvested colonies were confirmed as *Bartonella* by PCR and sequencing of *gltA* fragments. The overall prevalence of *Bartonella* in bats by culturing was 49.5% (105/212). *Bartonella* isolates were obtained from each of the eight sites. The prevalence of culture-positive bats varied from 16.7% at the Lavra site in Davit Gareja to 64.6% at Gliana Cave in Tskaltubo.

The range of prevalence varied from 16.7% in *P*. *pygmaeus* to 88.9% in *Mn*. *schreibersii*. The best model based on AICc included bat species only with a good amount of predictive power(AUC = 0.71) [49]. Based on the Wald test, there were significant differences among bat species (χ2 = 26.9, df = 6, p-value = 1.5E4) in *Bartonella* prevalence. Prevalence of *Bartonella* in *My*. *blythii* (odds ratio = 3.4, 95% CI = [1.1, 13], p-value = 0.044), *Mn*. *schreibersii* (odds ratio = 30.7, 95% CI = [6.9,188.4], p-value = 3.7E-5), and *R*. *euryale* (odds ratio = 9, 95% CI = [2.4, 40], p-value = 0.0017) was significantly higher.

### Coinfections

Culture observations from 16 bat samples revealed morphology differences among bacterial colonies. From these samples, *Bartonella*-like colonies were observed with morphologies that visually varied by size (small, large) and/or texture (rough, smooth). The number of visually different colonies per plate varied from one unique colony among TNTC similar colonies to an equal number of two unique colony morphologies. We did not attempt to estimate colony forming units (CFU) for individual bats suspected of coinfection. Sequencing analysis confirmed a coinfection with two different *Bartonella* sequences from these 16 samples (Table 1). Of those, seven were detected in *Mn*. *schreibersii*, three in *My*. *blythii*, one in *My*. *emarginatus*, two in *R*. *euryale*, and three in *R*. *ferrumequinum* (Table 1).

### Phylogeny based on *gltA* sequences

**Fig 1:**
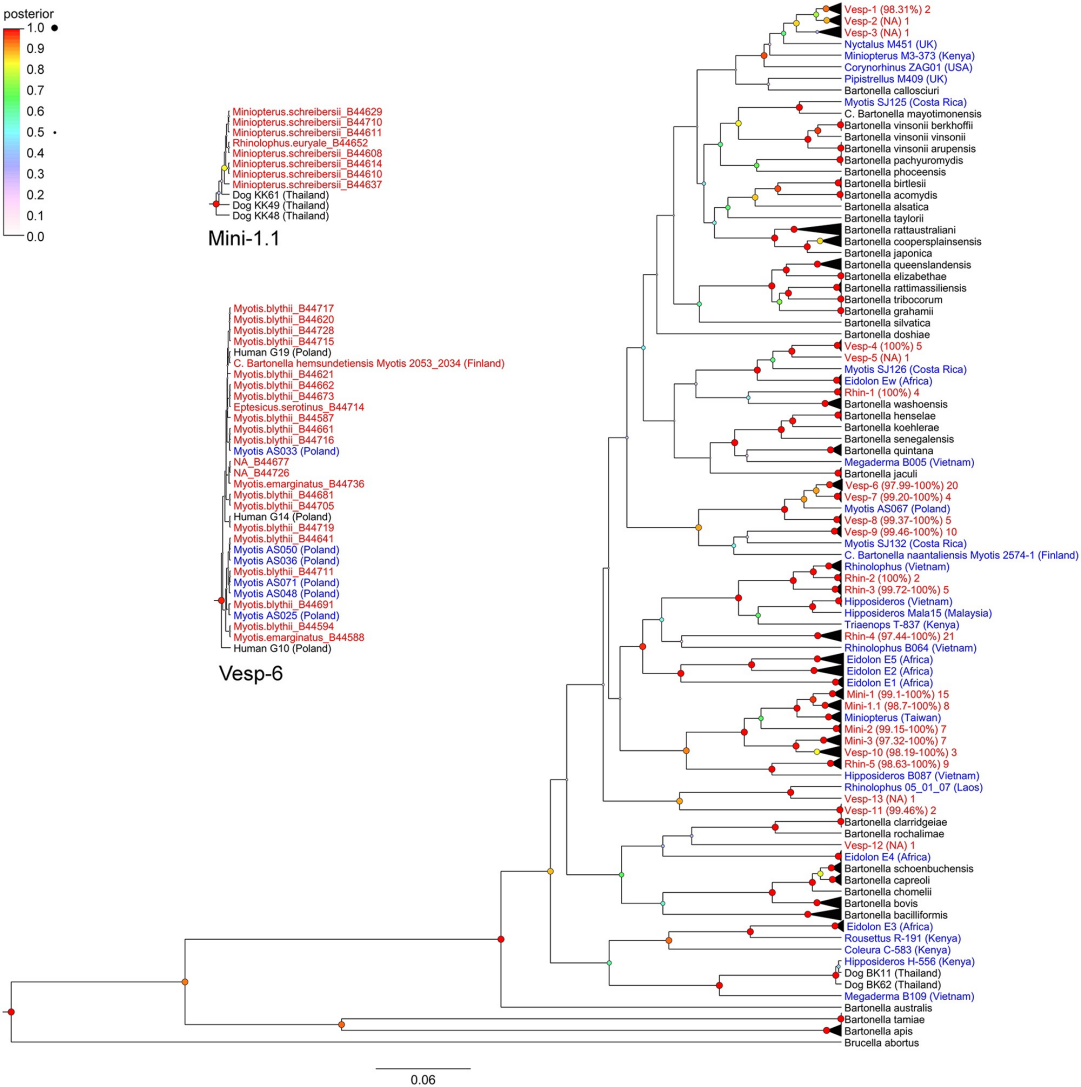
Phylogenetic relationships among citrate synthase (*gltA*) sequences from Georgian bats, other bat species, and known *Bartonella* species.

The Bayesian analysis indicated that most *gltA* sequences from Georgian bats cluster closely with eachother as distinct genogroups from known *Bartonella* species Based on a sequence identity threshold of 96%, we identified 22 distinct genogroups. Nucleotide sequence identity values varied between 97–100% within the identified genogroups. (Fig 1)

Results from BLAST searches for each *Bartonella* genogroup from Georgian bats are compiled in Table 3.

**Table 3:** BLAST search results for *gltA* sequences from each *Bartonella* genogroup from Georgian bats.

Georgian bats			Most similar sequence from GenBank			
Genogroup	Total	Per host (n)	GenBank accession number	Source	Location	Sequence nucleotide identity (%)
Mini-1	14	*Mn*. *schreibersii* (11)	HM545139, KT751153	*Miniopterus* sp., *Penicillidia leptothrinax* collected from *Miniopterus griveaudi*	Kenya, Madagascar	100, 96
		*E*. *serotinus* (1)				
		*My*. *blythii* (1)				
		*P*. *pygmaeus* (1)				
Mini-1.1	8	*Mn*. *schreibersii* (7)	FJ946852, JF500511	Dog, *Miniopterus schreibersii*	Thailand, Taiwan	99, 98
		*Rh*. *euryale* (1)				
Mini-2	7	*Mn*. *schreibersii* (7)	KT751143	*Penicillidia leptothrinax* collected from *Miniopterus aelleni*	Madagascar	98
Mini-3	6	*Mn*. *schreibersii* (6)	KT751152, FJ946854, HM545140	*Nycteribia stylidiopsis* collected from *Miniopterus gleni*, dog, *Miniopterus* sp.	Madagascar. Thailand, Kenya	100, 99, 99
Rhin-1	4	*R*. *ferrumequinum* (3)	AF470616	*Spermophilus beecheyi*	US	95
		*My*. *emarginatus* (1)				
Rhin-2	2	*R*. *ferrumequinum* (2)	KP100344, KP100345	*Rhinolophus sinicus*, *Rhinolophus acuminatus*	Vietnam	98, 97
Rhin-3	5	*My*. *blythii* (2)	KP100342, KP100344	*Rhinolophus sinicus*, *Rhinolophus acuminatus*	Vietnam	96, 95
		*R*. *euryale* (1)				
		*R*. *ferrumequinum* (2)				
Rhin-4	17	*R*. *euryale* (13)	JX416255, JX416239, KP100350	*Cyclopodia simulans* collected from *Ptenochirus jagori*, *Leptocyclopodia* sp. collected from *Harpionycteris whiteheadi*, *Rhinolophus acuminatus*	Philippines, Philippines, Vietnam	92, 92, 91
		*R*. *ferrumequinum* (3)				
		*Mn*. *schreibersii* (1)				
Rhin-5	9	*R*. *euryale* (6)	KP100355	*Hipposideros larvatus*	Vietnam	95
		*R*. *ferrumequinum* (3)				
Vesp-1	2	*My*. *emarginatus* (2)	KF003137, AJ871614	Bat flea collected from vespertilionid bat, *Pipistrellus* sp.	Finland, UK	99, 98
Vesp-2	1	*My*. *blythii* (1)	KF003122	*Myotis daubentonii*	UK	99
Vesp-3	1	*E*. *serotinus* (1)	KF003115, AJ871612	*Eptesicus nilssoni*, *Myotis mystacinus*	Finland, UK	99, 98
Vesp-4	5	*My*. *blythii* (5)	KJ816667	*Anatrichobius scorzai* collected from *Myotis keaysi*	Costa Rica	94
Vesp-5	1	*My*. *blythii* (1)	KJ816667	*Anatrichobius scorzai* collected from *Myotis keaysi*	Costa Rica	94
Vesp-6	18	*My*. *blythii* (15)	JQ695834, KR822802, HM116785	*Myotis myotis*, *Myotis daubentonii*, human	Poland, Finland, Poland	100, 99, 99
		*E*. *serotinus* (1)				
		*My*. *emarginatus* (2)				
Vesp-7	4	*E*. *serotinus* (1)	JQ695834, KR822802, HM116785	*Myotis myotis*, *Myotis daubentonii*, human	Poland, Finland, Poland	99, 97, 98
		*My*. *emarginatus* (3)				
Vesp-8	4	*My*. *blythii* (3)	JQ695834, KR822802, HM116785	*Myotis myotis*, *Myotis daubentonii*, human	Poland, Finland, Poland	96, 96, 96
		*My*. *emarginatus* (1)				
Vesp-9	8	*My*. *emarginatus* (6)	KF003129, KJ816689	*Myotis daubentonii*, *Basilia* sp. collected from *Myotis keaysi*	Finland, Costa Rica	93, 91
		*Mn*. *schreibersii* (1)				
		*My*. *blythii* (1)				
Vesp-10	3	*My*. *blythii* (3)	JX416246, JX416241, KT751152	*Basilia coronata* collected from *Tylonycteris* sp., *Basilia nattereri* collected from *Myiotis nattereri*, *Nycteribia stylidiopsis* collected from *Miniopterus gleni*	Malaysia, Slovenia, Madagascar	98, 98, 97
Vesp-11	2	*My*. *blythii* (2)	KT751154	*Penicillidia* cf. *fulvida* collected from *Miniopterus griveaudi*	Madagascar	92
Vesp-12	1	*My*. *emarginatus* (1)	KM030517, GU056189	*Eidolon helvum*, human	Africa, Thailand	91, 92
Vesp-13	1	*P*. *pygmaeus* (1)	KT751145, JX416252	*Penicillidia leptothrinax* collected from *Miniopterus manavi*, *Phthiridium* sp. *scissa* group collected from *Rhinolophus pearsoni*	Madagascar, Laos	97, 95

In some cases, Georgian bat sequences matched very closely with other *bartonella* sequences from related bats (same genus or family), but from distant locations. Other sequences, notably from genogroups Mini-1.1, Mini-3, and Vesp-6, clustered with *bartonella* sequences identified in dogs from Thailand [53] and in humans (forest workers) from Poland [54].

### Phylogeny based on multiple loci

**Fig 2:**
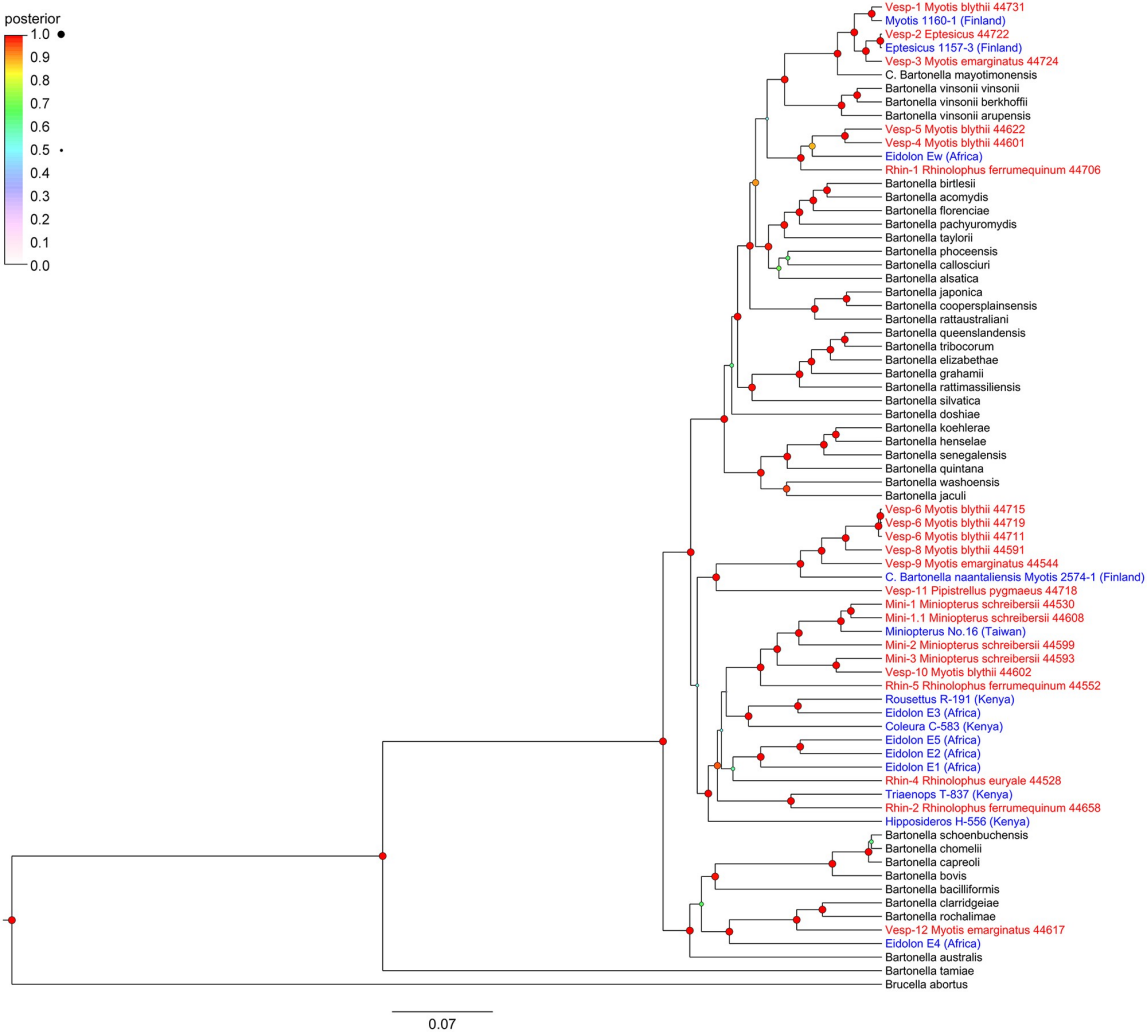
Phylogenetic relationships among *Bartonella* genogroups from Georgian bats, other genogroups from bats, and known *Bartonella* species using five genetic loci.

The phylogeny based on concatenated sequences from five genetic loci (*ftsZ*, *gltA*, *nuoG*, *rpoB*, and *groEL*) confirmed that most *Bartonella* genogroups from Georgian bats formed well-supported clades (posterior probability > 90%) with other *Bartonella* genogroups identified in bats. (Fig 2)

Genogroups Mini-1, Mini-1.1, Mini-2, Mini-3, Rhin-2, Rhin-4, Rhin-5, and Vesp-10 formed a well-supported clade with other *Bartonella* genogroups found in African pteropodid (*Eidolon helvum* and*Rousettus aegyptiacus*) [7,9], hipposiderid (*Hipposideros* sp. and *Triaenops persicus*) [7], andemballonurid (*Coleura afra*) [7] bats. Genogroups Mini-1 and Mini-1.1 clustered with another*Bartonella* genogroup found in *Miniopterus schreibersii* from Taiwan [13]. Genogroups Vesp-6, Vesp-8, Vesp-9, and Vesp-11 formed a second clade related to *Candidatus Bartonella naantaliensis* found in *Myotis daubentonii* from Finland [5]. These two clades were linked together by a node in the phylogeny; however, the posterior probability support for this node was only 53%.

**Fig 3:**
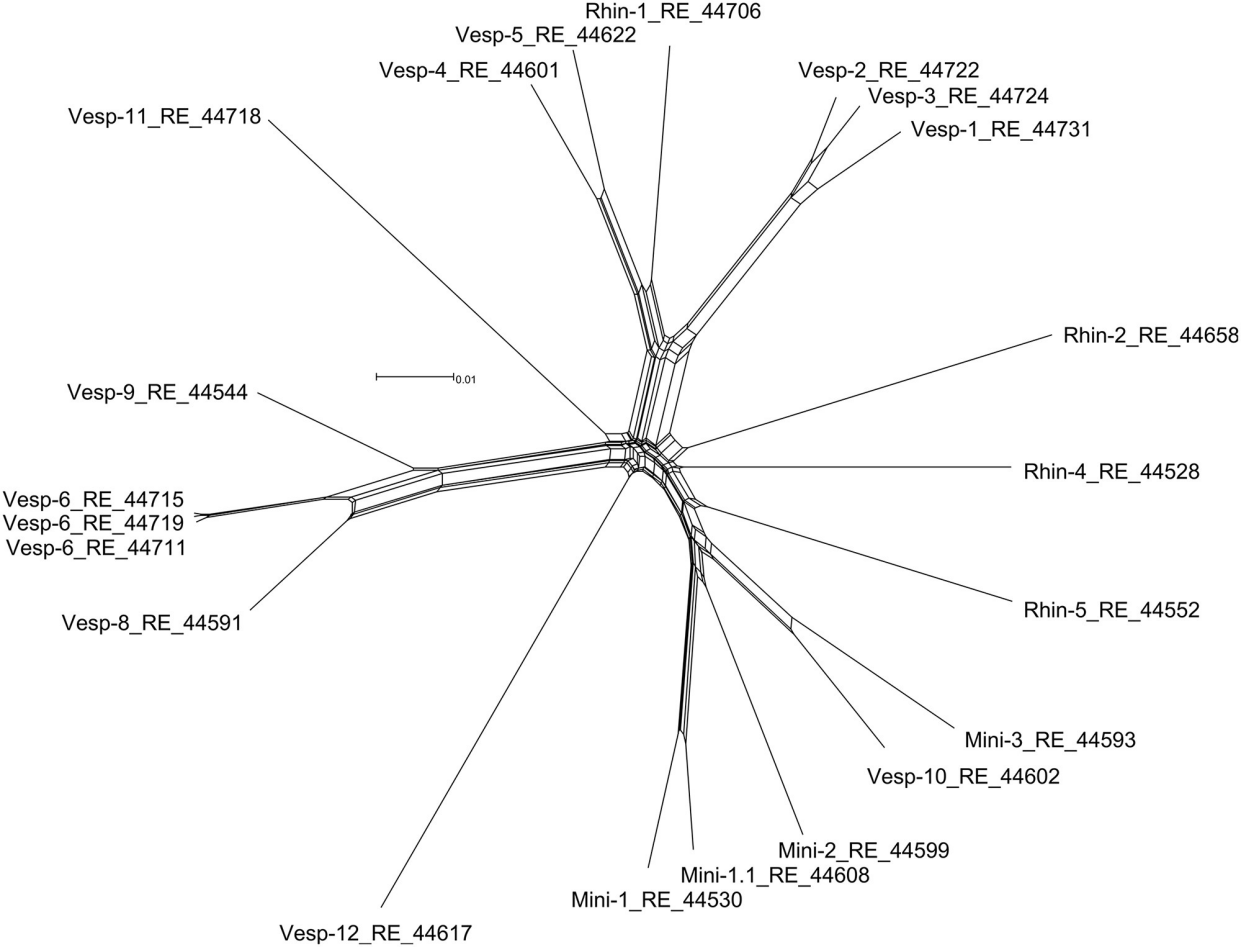
Network phylogeny of *Bartonella* genogroups isolated from Georgian bats.

Genogroups Rhin-1, Vesp-4, and Vesp-5 clustered with genogroup *Ew* from *Eidolon helvum* [7]. Genogroups Vesp-1, Vesp-2, and Vesp-3 clustered with *Bartonella mayotimonensis* isolated from a human endocarditis patient [26] and from European vespertilionid bats (*Eptesicus nilssonii* and *Myotis daubentonii*) [5]. These two clades were linked by a node, including *Bartonella vinsonii* subspecies, with low posterior probability support (50%). Finally, genogroup Vesp-12 clustered with genogroup *E4* from *Eidolon helvum* [9], as well as with *Bartonella clarridgeiae* and *Bartonella rochalimae*. The network phylogeny (Fig 3) indicated that most genogroups form distinct lineages, although there is some reticulation among related genogroups. In these cases, homologous recombination might be occurring among genogroups infecting a single bat species or a group of species. However, the pairwise homoplasy index [47] did not indicate significant evidence for recombination (mean = 0.6, variance = 1.7E-5, p-value = 0.5), suggesting that the reticulations in the network did not have a strong influence on the evolutionary history of these genogroups.

## Discussion

**Fig 4:**
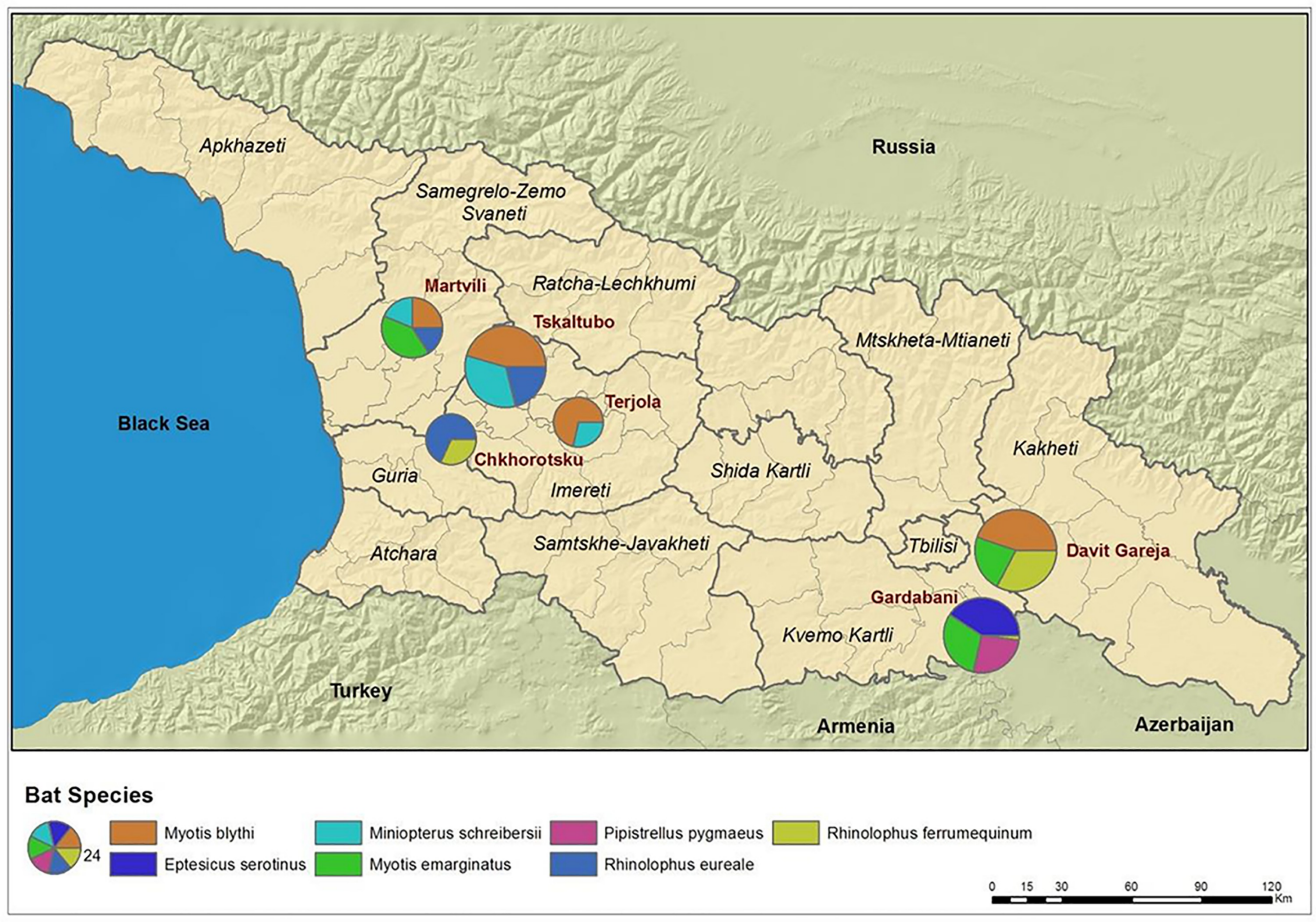
GIS map, sampling sites with bats species.

This report is the first to describe the prevalence, geographic patterns, and genetic characteristics of*Bartonella* species found in bat communities within the southern Caucasus. Several interestingconclusions can be drawn from the study. First, we provided the evidence that *Bartonella* infections arewidespread and highly prevalent in all seven bats species tested. This observation is comparable to the investigations of *Bartonella* species in bats from other geographic regions (e.g., Kenya, Guatemala, and Peru) where high prevalence and diversity of *Bartonella* strains have been reported [7,15,16]. However, in our study the prevalence of infection varied greatly between bat species (nearly 89% in *Mn*. *schreibersii* and below 17% in *P*. *pygmaeus*) as well as between study sites. The difference inprevalence between locations can be likely explained by bat community composition (Table 1). For example, *P*. *pygmaeus* was only captured at one location whereas *Mn*. *schreibersii* was collected from many sites, and the bat colony at John the Baptist Cave in Davit Gareja consisted solely of *My*. *blythii*. (Fig 4).

These sampling biases should be considered when interpreting *Bartonell*a prevalence values. We alsocannot exclude other factors, including the level of ectoparasite infestation in bats that may influence theprevalence of *Bartonella* in each bat species and locations.

We observed several coinfections among sampled bats. The phenomenon of coinfections with two or three different *Bartonella* species or genotypes in blood has been described previously for rodents [55]. Interestingly, a high rate of coinfection was observed in one particular bat species, *Mn*. *schreibersii*. Seven of the 27 (26%) *Mn*. *schreibersii* tested were coinfected with two different *Bartonella* genotypes (Patterns of codivergence of *Bartonella* with their bat hosts have varied among studies and aroundthe world [7,15,16,20]. For *Bartonella* genogroups found in Georgian bats, some general patterns of hostspecificity at the genus and family level are apparent. Nearly all of the isolates (33/35) from *Mn*.*schreibersii* aligned with genogroups Mini-1, Mini-1.1., Mini-2, or Mini-3 (Table 3). Based on sequence identity at the *gltA* gene, all of these genogroups closely matched *Bartonella* sequences from other*Miniopterus* spp. (e.g., *Mn*. *griveaudi*, *Mn*. *aelleni*, and *Mn*. *gleni*) from Madagascar [11]. Thirty-seven of 38 isolates obtained from *Rhinolophus* spp. (*R*. *euryale* or *R*. *ferrumequinum*) belonged to genogroupsRhin-1, Rhin-2, Rhin-3, or Rhin-4. Genogroups Rhin-2 and Rhin-3 cluster with *Bartonella* sequences identified in *R*. *acuminatus* and *R*. *sinicus* from Vietnam [14]. Most isolates (54/60) obtained from vespertilionid bats (*Eptesicus*, *Myotis*, and *Pipistrellus* spp.) were members of genogroups Vesp-1 to Vesp-12 with closely matching sequences found in other vespertilionid bats [4–6,17,56].

Despite these general host associations, specificity of genogroups at the genus or family levelwas not strict, with some instances of apparent spillover of *Bartonella* into atypical hosts. For example, isolates of *Bartonella* from genogroup Mini-1 were found in *E*. *serotinus*, *My*. *blythii*, and *P*. *pygmaeus*, and isolates of *Bartonella* from genogroups Rhin-1 and Rhin-3 were found in *My*. *emarginatus* and *My*. *blythii*, respectively (Table 3). Though infrequent, these spillover events can be explained by the co-occurrence of these bat species in the same roosts (Table 1), wherein transmission may be facilitated by shared vectors. Ectoparasites were collected from bats at the sampled sites in Georgia in 2012, but have not yet been identified and are thus not included in this study. However, there are numerous ectoparasite species reported on our seven focal bat species in the literature. While some ectoparasite species preferentially feed on specific bat hosts, they can also be found infrequently on other bat hosts, which may lead to transmission of bacteria. For example, bat flies (Diptera: Nycteribiidae) can be closely associated with one or a few bat hosts: *Basilia nana* with *Myotis bechsteinii* [57], *Basilia nattereri* with *Myotis nattereri* [58], *Nycteribia schmidlii* and *Penicillidia conspicua* with *Miniopterus schreibersii* [59], and *Phthiridium biarticulatum* with *Rhinolophus* spp. [60]. Nevertheless, there are recorded incidents of these bat flies on other bat hosts, including the focal species in this study: *Basilia nana* recorded on *My*. *blythii* and *My*. *emarginatus* [61], *Basilia nattereri* recorded on *E*. *serotinus* [62], *Nycteribia schmidlii* recorded on *My*. *blythii*, *My*. *emarginatus*, *R*. *euryale*, and *R*. *ferrumequinum* [61,63], *Penicillidia conspicua* on *My*. *blythii* [61], and *Phthiridium biarticulatum* on *E*. *serotinus*, *Mn*. *schreibersii*, *and My*. *emarginatus* [61,64]. Other ectoparasites can have broader and more evenly distributed host ranges, and may be found infesting our focal bat species. *Argas vespertilionis* (Ixodida: Argasidae) has been collected from *E*. *serotinus*, *My*. *blythii*, *P*. *pygmaeus*, and *R*. *ferrumequinum* [61,65,66]. *Cimex pipistrelli* (Hemiptera: Cimicidae) has been reported parasitizing *E*. *serotinus*, *My*. *blythii*, *My*. *emarginatus*, *P*. *pygmaeus*, and *R*. *ferrumequinum* [67,68]. Additionally, *Spinturnix myoti* (Mesostigmata: Spinturnicidae) has been recorded on *E*. *serotinus*, *Mn*. *schreibersii*, *My*. *blythii*, *R*. *euryale*, and *R*. *ferrumequinum* [69–71]. This short review of the literature is not exhaustive, but is meant to illustrate that nonspecific parasitism by *Bartonella* genogroups in some bat hosts can potentially be explained by sharing of ectoparasites. Future analyses exploring the influence of ectoparasite distributions on sharing of *Bartonella* genogroups among bats are in progress.

The sequence characterization of five house-keeping genes (*ftsZ*, *gltA*, *nuoG*, *rpoB*, and *groEL*) along with the network phylogenetic analysis strongly indicated that many genogroups characterized in our study can be segregated into new *Bartonella* species according to established demarcationcriteria considering loci separately [38],with sequence identity >95% based on concatenated loci for most pairwise comparisons within each *Bartonella* genogroup. The host associations observed for most of identified genetic clusters also supports the biological basis for discrimination of the species. As was reasoned previously [72], a refined approach that combines data from multiple genetic markers with ecological information about host specificity provides more reliable and tangible demarcations of *Bartonella* species compared to sequence analysis alone. For example, genogroups Vesp-1, Vesp-2, and Vesp-3 share 92%, 93%, and 92% nucleotide identity, respectively, with *Bartonella mayotimonensis*, the bacterial species discovered in a human patient in the United States [26]. However, *B*. *mayotimonensis* is closest (95%) at the *gltA* locus to a sequence identified in a bat fly *Anatrichobius scorzai* taken from a bat *Myotis keaysi* in Costa Rica [17]. It is likely that clusters Vesp-1, Vesp-2, Vesp-3, and the bat fly strain from Costa Rica can be assigned to the *B*. *mayotimonensis* species, but using the *gltA* locus alone creates an artifactual split among the genogroups. When all five concatenated loci were considered, genogroups Vesp-1, Vesp-2, and Vesp-3 shared pairwise sequence identities between 96.9–98.11%. Considering their relatedness and apparent specificity to vespertilionid bats (*Eptesicus*, *Myotis*, and *Pipistrellus* spp.) [5], all of these genogroups may be included as one species. The pairwise identities of these genogroups with *B*. *mayotimonensis* ranged 95.1–95.5%, which is near the previously established minimum threshold for distinguishing between *Bartonella* species (95.4% for *rpoB* sequences [38]) and we argue it should be considered synonymous with Vesp-1, Vesp-2, and Vesp-3. Similarly, genogroups Vesp-6 and Vesp-8 were 95.9% identical and considering their apparent specificity to vespertilionid bats (*Eptesicus* and *Myotis*) [5] they may also constitute a single *Bartonella* species. This is also true for genogroups Vesp-4 and Vesp-5 found in one bat species, *My*. *blythii* (96.3% sequence identity) and genogroups Mini-1 and Mini-1.1 found in *Mn*. *schreibersii* (96.6% sequence identity).

The most intriguing and important results from this study is the identification of bat-borne*Bartonella*, which are similar to *Bartonella* strains previously reported in humans and in dogs. Thepublic health relevance of bat-borne *Bartonella* infection has been discussed since the identification ofsuch bacteria in bats from Kenya [7]. Our results highlight the importance of *Bartonella* surveillance inbats, as it can help to identify potential wildlife reservoirs of human cases. Although some sequences of *Bartonella* found in Georgian bats clustered with *B*. *mayotimonensis*, the genetic distances were relatively long, as noted above. We might speculate that *Bartonella* more closely related to thishuman case are circulating in vespertilionid bats in the North and South America rather than in Europe. Even more unexpected was the discovery of *Bartonella* strains in Georgian bats which wereidentical or very similar to ones reported in forest workers from Poland. The study in Poland wasconducted to evaluate the level of exposure of 129 forest workers to diverse tick-borne pathogens [54].*Bartonella* antibodies were reported in about 30% of tested individuals, but more importantly, threeserologically-positive samples were also positive for *Bartonella* nucleic acids by PCR and sequencing. The *gltA* sequences identified in that study were distinct from all previously reported. They were closest (90% similarity) to *B*. *koehlerae*, *B*. *clarridgeiae* and a genotype from an arthropod from Peru. They were deposited in GenBank (accessions HM116784, HM116785, and HM116786) as uncultured *Bartonella* spp. [54]. All strains identified in our study as genotype Vesp-6 were 100% identical by *gltA* sequences to the HM116785 sequence. Vesp-6 is the largest genogroup found in bats from Georgia containing 18 sequences from *My*. *blythii* (n = 15), *My*. *emarginatus* (n = 2), and *E*. *serotinus* (n = 1). All of these bat species are listed as occurring in southern Poland where the investigation of forest workers was conducted [73–75].

Another surprising discovery was that *Bartonella* strains observed in this study were closely related to those identified in stray dogs from Thailand., Bai et al. [53] provided evidence of common *Bartonella* infections and diverse *Bartonella* species in the blood of stray dogs from Bangkok and Khon Kaen (northeastern province of Thailand). Besides two *Bartonella* species (*B*. *elizabethae* and *B*. *taylorii*) detected in stray dogs from Khon Kaen, the authors also reported two genotypes (KK20 and KK61) that could potentially represent a new species [53]. Performing the analysis of *Bartonella* strains found in bats from Georgia, we found that sequences of the strains from genogroup Mini-1.1 obtained from *Mn*. *schreibersii* (n = 7) and *R*. *euryale* (n = 1) were 99% similar to those dog sequences from Thailand (strain KK61, GenBank accession FJ946852). Likewise, seven sequences from *Mn*. *schreibersii* (genogroup Mini-3) were 99% similar to the sequences of the strain KK20 from stray dogs from Khon Kaen, Thailand (GenBank accession FJ946854). Bat species belonging to the genus *Miniopterus* (e.g., *Mn*. *magnater* and *Mn*. *pusillus*) are present in Thailand [76].

The identification of diverse *Bartonella* strains in Georgian bats, which are identicalor similar to the strains previously described in humans and in companion animals in other geographic areas grants special attention in future studies to evaluate their role as potential zoonotic agents. Aparticular question remains regarding the route of transmission of bat-associated *Bartonella* to people. Itis easier to speculate how stray dogs, which may scavenge for grounded bats, can become infected withbat-associated *Bartonella*, but the question concerning transmission of bat-borne strains to humans ismore challenging [77]. However, the human case of endocarditis linked to a bat-associated *Bartonella*species [5,26] suggests that such transmission can occur. Some bat ectoparasites are known tooccasionally bite humans, including *Argas vespertilionis* and *Cimex pipistrelli* [78–80]. Thus, *Bartonella* surveillance should include not only mammals, but also their vectors whenever possible to better understand the risks of disease transmission.

## Supporting information

S1 Appendix (DOCX)
