# Supplementary material for: Prevalence, diversity, and host associations of Bartonella strains in bats from Georgia (Caucasus)
Source: PLoS Negl Trop Dis. 2017 Apr 11;11(4):e0005428. doi: 10.1371/journal.pntd.0005428 (PMC5400274; doi:10.1371/journal.pntd.0005428)
Supplement: S1 Appendix — (DOCX) [file pntd.0005428.s001.docx]

**Appendix**

**Additional details of phylogenetic analyses**

For all phylogenetic reconstructions using BEAST v1.8.3 [1,2] on the five genetic loci analyzed (*ftsZ*, *gltA*, *groEL*, *nuoG*, and *rpoB*), the following priors were used for the GTR+Γ+I model, suggested as default weak priors in BEAST:

A-C substitutions ~ Gamma(0.05, 10), initial value = 1

A-G substitutions ~ Gamma(0.05, 20), initial value = 1

A-T substitutions ~ Gamma(0.05,10), initial value = 1

C-G substitutions ~ Gamma(0.05,10), initial value = 1

G-T substitutions ~ Gamma(0.05,10), initial value = 1

Gamma shape parameter ~ Exponential(0.5), initial value = 0.5

Proportion of invariant sites ~ Uniform(0, 1), initial value = 0.5

**Additional details of statistical analyses**

Logistic model selection was performed using the ‘dredge’ function in the R package ‘MuMIn’ [3,4] using Akaike’s information criterion corrected for finite sample sizes (AICc) [5]. Models were considered equally favored if the difference in AICc from the top model was less than two [6]. The Wald test of fixed effects was performed on top models using the function ‘wald.test’ in the R package ‘aod’ [7]. The ratio of model deviance to residual degrees of freedom was checked and half-normal plots were made using the ‘halfnorm’ function of ‘faraway’ [8]. The area under each model’s receiver operating characteristic curve (AUC) was calculated using the ‘performance’ function in the package ‘ROCR’ [9]. Model predictions were considered good if AUC > 0.7 [10].

**References**

1. Drummond AJ, Rambaut A (2007) BEAST: Bayesian evolutionary analysis by sampling trees. BMC Evol Biol 7: 214. Available: http://bmcevolbiol.biomedcentral.com/articles/10.1186/1471-2148-7-214.

2. Drummond AJ, Suchard MA, Xie D, Rambaut A (2012) Bayesian phylogenetics with BEAUti and the BEAST 1.7. Mol Biol Evol 29: 1969–1973. Available: http://mbe.oxfordjournals.org/cgi/doi/10.1093/molbev/mss075.

3. Bartoń K (2016) MuMIn: multi-model inference. Available: http://cran.r-project.org/package=MuMIn.

4. R Core Team (2015) R: a language and environment for statistical computing. R Found Sttistical Comput Vienna, Austria. Available: http://www.r-project.org.

5. Burnham K, Anderson D (2004) Multimodel inference: understanding AIC and BIC in model selection. Sociol Methods Res 33: 261–304.

6. Burnham K, Anderson D (2002) Model selection and multimodel inference: a practical information-theoretic approach, second edition. New York: Springer. 488 p. Available: http://linkinghub.elsevier.com/retrieve/pii/S0304380003004526.

7. Lesnoff M, Lancelot R (2012) aod: analysis of overdispersed data. Available: https://cran.r-project.org/package=aod.

8. Faraway J (2016) faraway: functions and datasets for books by Julian Faraway. Available: https://cran.r-project.org/package=faraway.

9. Sing T, Sander O, Beerenwinkel N, Lengauer T (2015) ROCR: visualizing the performance of scoring classifiers. Available: https://cran.r-project.org/package=ROCR.

10. Hosmer DW, Lemeshow S (2000) Applied logistic regression, second edition. New York: Wiley and Sons, Inc. doi:10.1002/0471722146.
